# Very Long-Chain Acyl-CoA Dehydrogenase Deficiency: High Incidence of Detected Patients With Expanded Newborn Screening Program

**DOI:** 10.3389/fgene.2021.648493

**Published:** 2021-04-27

**Authors:** Ziga I. Remec, Urh Groselj, Ana Drole Torkar, Mojca Zerjav Tansek, Vanja Cuk, Dasa Perko, Blanka Ulaga, Neza Lipovec, Marusa Debeljak, Jernej Kovac, Tadej Battelino, Barbka Repic Lampret

**Affiliations:** ^1^Clinical Institute for Special Laboratory Diagnostics, University Children’s Hospital, University Medical Centre Ljubljana, Ljubljana, Slovenia; ^2^Department of Endocrinology, Diabetes and Metabolic Diseases, University Children’s Hospital, University Medical Centre Ljubljana, Ljubljana, Slovenia; ^3^Faculty of Medicine, University of Ljubljana, Ljubljana, Slovenia; ^4^Unit for Clinical Dietetics, University Children’s Hospital, University Medical Centre Ljubljana, Ljubljana, Slovenia

**Keywords:** VLCAD deficiency, VLCADD, neonatal screening, NBS, MS/MS, NGS, *ACADVL* gene, acylcarnitines

## Abstract

Very long-chain acyl-CoA dehydrogenase deficiency (VLCADD) is a rare autosomal recessive disorder of fatty acid metabolism with a variable presentation. The aim of this study was to describe five patients with VLCADD diagnosed through the pilot study and expanded newborn screening (NBS) program that started in 2018 in Slovenia. Four patients were diagnosed through the expanded NBS program with tandem mass spectrometry; one patient was previously diagnosed in a pilot study preceding the NBS implementation. Confirmatory testing consisted of acylcarnitines analysis in dried blood spots, organic acids profiling in urine, genetic analysis of *ACADVL* gene, and enzyme activity determination in lymphocytes or fibroblasts. Four newborns with specific elevation of acylcarnitines diagnostic for VLCADD and disease-specific acylcarnitines ratios (C14:1, C14, C14:2, C14:1/C2, C14:1/C16) were confirmed with genetic testing: all were compound heterozygotes, two of them had one previously unreported *ACDVL* gene variant each (NM_000018.3) c.1538C > G; (NP_000009) p.(Ala513Gly) and c.661A > G; p.(Ser221Gly), respectively. In addition, one patient diagnosed in the pilot study also had a specific elevation of acylcarnitines. Subsequent *ACDVL* genetic analysis confirmed compound heterozygosity. In agreement with the diagnosis, enzyme activity was reduced in five patients tested. In seven other newborns with positive screening results, only single allele variants were found in the *ACDVL* gene, so the diagnosis was not confirmed. Among these, two variants were novel, c.416T > C and c.1046C > A, respectively (p.Leu139Pro and p.Ala349Glu). In the first 2 years of the expanded NBS program in Slovenia altogether 30,000 newborns were screened. We diagnosed four cases of VLCADD. The estimated VLCADD incidence was 1:7,500 which was much higher than that of the medium-chain acyl-CoA dehydrogenase deficiency (MCADD) cases in the same period. Our study also provided one of the first descriptions of *ACADVL* variants in Central-Southeastern Europe and reported on 4 novel variants.

## Introduction

Very long-chain acyl-CoA dehydrogenase deficiency (VLCADD, OMIM 201475) is the second most common disorder of inborn errors of fatty acid metabolism; its incidence varies between 1:30,000 and 1:400,000 live births, with some outliers such as Saudi Arabia with reported incidence of 1:3200 and Taiwan with 1:1,400,000. In Europe incidence range from 1:77,000 in Germany and Netherlands to 1:400,000 in Czech Republic (Arnold et al., 2009; Spiekerkoetter et al., 2009; Lindner et al., 2010; Marsden et al., 2021). Incidence of VLCADD increased after the introduction of an expanded newborn screening program with the use of tandem mass spectrometry (MS/MS) allowing early detection of patients (Boneh et al., 2006; Merritt et al., 2014). VLCADD is caused by pathogenic variants in the *ACADVL* gene and is inherited in an autosomal recessive manner, resulting in deficient enzyme in the mitochondrial β-oxidation of long-chain fatty acids. Fatty acids are an important source of energy during prolonged fasting, physical exercise, and febrile infections when the body requires more energy. In VLCADD long-chain fatty acids with chain lengths of 14–20 carbons are not metabolized, which can lead to metabolic crises due to inadequate energy supply. This lack of energy may result in symptoms such as lethargy and hypoglycemia. Fats that are not properly broken down can also build-up and damage tissues in the heart, liver, and skeletal muscles, which can cause the other clinical features observed in people with VLCADD [Leslie et al., 1993; Very Long Chain Acyl CoA Dehydrogenase Deficiency (VLCADD) - NORD (National Organization for Rare Disorders)].

Clinical presentations in patients are very heterogeneous, with three major phenotypes. Severe infantile VLCADD has an early onset, usually within the first months of life. It has high mortality and a high incidence of hypoketotic hypoglycemia, liver disease, cardiac arrhythmias, cardiomyopathy, and pericardial effusion. The moderate VLCADD has a later onset (late infancy to early childhood) and usually presents with lower mortality, hypoketotic hypoglycemia, hepatomegaly, and rarely cardiomyopathy. The mild or late-onset VLCADD presents in older children and young adults (usually > 10 years of age) with isolated skeletal muscle involvement, exercise intolerance, myalgia, rhabdomyolysis, and myoglobinuria usually triggered by exercise, fasting, or stress, but viral infection can also precipitate this presentation. In rare cases, it can lead to renal failure and can be fatal. Some patients presenting with myopathic disease may have a history of hypoglycemia in infancy or childhood (Leslie et al., 1993; Vianey-Saban et al., 1998; Very Long Chain Acyl CoA Dehydrogenase Deficiency (LCAD) – National Organization for Rare Disorders [NORD], 2020).

Optimal VLCADD management requires ongoing assessment of clinical and nutritional status. Since nutritional intervention is a cornerstone in treating VLCADD, close collaboration with metabolic clinical dietitian is crucial to improve patients’ clinical outcomes. The goals of nutrition therapy are to minimize the production of abnormal fatty acid metabolites with long-chain triglycerides (LCT) restriction and prevention of metabolic crises due to a lack of an adequate energy supply. Nutrition therapy is tailored individually, dependent on the severity of the disorder and patients’ age and typically includes restriction of dietary intake of LCT together with supplementation with medium-chain triglycerides (MCT) (Van Calcar et al., 2020).

VLCADD can be detected with the accumulation of characteristic acylcarnitines in blood only a few days after birth. The specific acylcarnitine profile can be accurately measured in newborn dried blood spots (DBS) by MS/MS (Wilcken et al., 2003). The primary marker is elevated C14:1 acylcarnitine (tetradecenoylcarnitine), together with other long-chain acylcarnitines and disease-specific acylcarnitines ratios that can be calculated (C14, C14:2, C14:1/C2, C14:1/C16). As previously reported, MS/MS has a high false-positive rate and it is often difficult to differentiate between true positives, heterozygous carriers, and false positives (Spiekerkoetter, 2010; Bo et al., 2020). False positives can also occur due to insufficient breastfeeding, which might cause an elevation of C14:1 and C14:1/C2. That’s why it is suggested that in suspected VLCADD, attending doctors should pay attention to body weight changes recorded during newborn body mass screening (Bo et al., 2020).

On the other hand, the second DBS sample for acylcarnitine measurement could give normal results even if disease is present, due to the switch to anabolic condition at the time of the second sampling (Boneh et al., 2006; Spiekerkoetter, 2010; Hesse et al., 2018). NGS could be of great help in confirmatory diagnostics; however, it is inconclusive in cases of novel genetic variants, and variants of unknown significance (VUS) (Hoffmann et al., 2012). Despite the abovementioned limitations, we decided to introduce next-generation sequencing (NGS) for confirmatory testing of positive NBS results (Smon et al., 2018; Lampret et al., 2020). In the case of positive NGS, enzyme testing in fibroblasts and lymphocytes as well as flux studies in fibroblasts have to be performed (Diekman et al., 2015; Hesse et al., 2018; Bleeker et al., 2019; Rovelli et al., 2019).

This study aimed to describe five patients with VLCADD diagnosed through the pilot and expanded newborn screening (NBS) program in Slovenia (from 2018).

## Materials and Methods

Dried blood samples were taken 48–72 h after the birth. Acylcarnitines were analyzed from 3 mm disk of DBS with MS/MS (Xevo TQD, Waters, Milford, Massachusetts, United States) using NeoBase^®^ 2 Non-derivatized MSMS kit (Perkin Elmer, Waltham, Massachusetts, United States) which quantifies the tested metabolites with stable-isotope-labeled internal standards and allows the detection of numerous metabolites simultaneously in a single run. The sample preparation was based on extraction without derivatization. Positive electrospray ionization and multiple reaction monitoring (MRM) mode were used for MS/MS.

For NGS confirmatory testing, an in-house panel of 72 genes was developed (Supplement 1; Lampret et al., 2020). The NGS panel was designed around the core of causative genes for the 18 diseases included in the current NBS program. Additional genes for the conditions that present with the increase of the same metabolites as the targeted disease were added, to aid differential diagnostics (Šmon et al., 2015; Lampret et al., 2020). Variants detected with NGS with vertical coverage below 100× were additionally confirmed by Sanger sequencing. For genetic analysis, whole blood samples were used for isolation of genomic DNA with established laboratory protocols based on FlexiGene DNA isolation kit (Qiagen, Hilden, Germany). For genetic analysis, NGS was performed using MiSeq desktop sequencer coupled with MiSeq Reagent kit v3 (both Illumina, San Diego, United States). The regions of interest were enriched using Agilent SureselectXT Target Enrichment System (Agilent Technologies Inc., Santa Clara, CA, United States) following the manufacturer’s instructions. We reached at least 10× coverage for 99.9% of regions of interest for each patient. Genetic testing of *ACADVL* gene of Patient 1 was performed at Laboratory Genetic Metabolic Diseases, Emma Chirdren’s Hospital AMC.

Urine organic acids were measured with an in-house method (Lampret et al., 2015). Briefly, to 1 mL of urine, we added O-ethylhydroxylamine hydrochloride (Sigma-Aldrich, Munich, Germany) and incubated the solution for 15 min at room temperature. 2-phenylbutyric acid (Sigma-Aldrich, Munich, Germany) was added as an internal standard in the concentration of 100 mmol per mol of creatinine. Urine was acidified and then the solution was saturated with NaCl (Sigma-Aldrich, Munich, Germany). After the addition of ethylacetate (Sigma-Aldrich, Munich, Germany) the solution was vortexed and centrifuged. The organic layer was transferred to a clean glass tube, and the ethylacetate evaporated under a stream of nitrogen. Finally, pyridine (Fluka, Buchs, Switzerland) and N, O-bis(trimethylsilyl)trifluoroacetamine (Sigma-Aldrich, Munich, Germany) were added, the solution mixed and analyzed with Agilent 5975C Series GC/MSD (Agilent Technologies, Santa Clara, CA, United States) on Agilent Ultra2 column (Agilent Technologies, Santa Clara, CA, United States).

If the second DBS card was collected before 10 days after birth, the same method as for the first sample was used (NeoBaseTM 2). When the second sample was collected later than that, confirmatory testing was performed with the Chromsystems method and different reference ranges were applied. Acylcarnitines for confirmatory testing were analyzed from dried blood spots and derivatized with Chromsystems kit MassChrom^TM^ Amino Acids and Acylcarnitines from Dried Blood (Chromsystems Instruments & Chemicals GmbH, Grüfelfing/Munich, Germany). The sample preparation was based on extraction from a 3 mm disk of DBS followed by derivatization to butyric esters. Positive electrospray ionization and multiple reaction monitoring (MRM) mode were used. They were quantified by PerkinElmer 200 HPLC system (Perkin Elmer, Waltham, Massachusetts, United States) coupled to AB Sciex 3200 QTRAP (AB SCIEX, Singapore).

Enzymatic activity of VLCAD was determined using the UPLC-UV method at AMC Amsterdam UMC, Amsterdam on lymphocytes from whole blood samples and in one case from cultured fibroblasts (Amsterdam UMC and Locatie AMC, 2021 - Very long-chain acyl-CoA dehydrogenase (VLCAD)).

To assess the adequacy of the patient’s diet, their caregivers were asked to record the intake of all foods, drinks, and food supplements consumed over 3 consecutive days. The average values of recording for all 3 days together were calculated and compared with recommendations for energy, total fat, long-chain fat, medium-chain fat, linoleic acid, and alpha-linolenic acid intake (Table 1) by an experienced clinical dietitian. For the nutritional analysis, we used the Prodi 6.10 Expert plus software (Nutri-Science, Stuttgart, Germany, 2020), which contains the database of approximately 15,000 foods from the Bundeslebensmittelschlüssel 3.01 (BLS 3.01) database, Fachmann-Kraut-Nährwerttabellen (FKN, Stuttgart, 2005) database, and industrial products and dietetic foods.

**TABLE 1:** Characteristics of nutritional management of VLCADD patients.

		**Calculated actual intake^+^**							**Recommended intake**						
Patient*	**Type**	**Energy**		**Total fat**	**Long- chain fat**	**Medium- chain fat**	**Linoleic acid**	**alpha-Linolenic acid**	**Energy^#^**		**Total fat^$^**	**Long-chain fat^$^**	**Medium-chain fat^$^**	**Linoleic acid^$^**	**Alpha-Linolenic acid^$^**
		**kcal/day**	**kcal/kg**	**% of total energy**					**kcal/day**	**kcal/kg**	**% of total energy**				
1	Moderate	1267	51	25	9	16	3	0.5	1530	67	25–35	15–25	10–20	3–6	0.5–1.2
2	Moderate	958	88	31	16	15	4	0.6	770	80	30–40	20–30	10–20	3–6	0.5–1.2
3	Moderate	994	83	33	20	13	4	0.6	720	80	30–40	20–30	10–20	3–6	0.5–1.2
4	Severe	997	110	31	10	21	5	0.5	770	80	30–40	10–15	10–30	3–6	0.5–1.2

*Actual intake is based on dietary records of 3 consecutive days calculated by Prodi 6.10 Expert plus Nutri-Science software (Stuttgart, Germany, 2020). *Patient 5 is not included in the table as she does not yet have special dietary restrictions except for fasting precautions. ^#^Average daily energy requirements in clinical practice adapted from: chapter 1, table 1.12 page 11 (Shaw, 2020). ^$^Recommended fat (total, long-chain and medium chain) intake for individuals with VLCAD when well adopted from Van Calcar et al. (2020).

Informed consent for genetic testing and anonymous presentation of clinical data was obtained from parents of all patients that underwent confirmatory NGS, and all analyses were performed as a part of a diagnostic procedure according to the principles of the Helsinki Declaration (WMA, 2020 WMA Declaration of Helsinki—Ethical Principles for Medical Research Involving Human Subjects—WMA—The World Medical Association). The studies involving human participants were reviewed and approved by The National Medical Ethics Committee (KME: 56/01/14).

### Case Series

**Patient 1** is a 7-year-old girl, who was first seen by our NBS team at 9.5 months after screened positive on a pilot metabolic screening project. She is the first child of non-consanguineous parents. The paternal grandfather had hypertrophic cardiomyopathy. She was born at 41-weeks of gestation, with appropriate birth measures. In the asymptomatic state, ammonia was up to 90 μmol/L (10–47 μmol/L), and creatine kinase (CK) 2.52 μkat/L (<2.50 μkat/L). VLCADD was confirmed by low enzyme activity (Table 2) and analysis of the *ACADVL* gene (Table 3). She has had no metabolic crisis to date. She had a single episode of leukocytopenia and thrombocytopenia, which resolved spontaneously. Echocardiogram (ECHO) of the heart was performed every 2 years and revealed no pathological findings. There has been no organomegaly on the abdominal ultrasound (US). Growth parameters and developmental assessment have been in the normal range. The patient was exclusively breastfed for 3 weeks and then, due to an insufficient weight gain, breastfeeding was supplemented with the regular milk formula. A LCT restricted diet was introduced at the age of 2 years. The intake of LCT was limited to 20–35% of total energy (Table 1), according to the recommendations (Van Calcar et al., 2020). Dietary requirements for essential fatty acids are being met with walnut oil and docosahexaenoic acid (DHA) supplementation.

**TABLE 2:** Comparison of biochemical characteristics of confirmed VLCADD patients, heterozygous *ACADVL* variant carriers and those who were negative at re-testing at NBS.

	Subject*		**P1**	**P2**	**P3**	**P4**	**P5**	**P6**	**P7**	**P8**	**P9**	**P10**	**P11**	**P12**	**P13**	**P14**	**P15**	**P16**	**P17**	**P18**
			**VLCADD positive patients**					**VLCADD negative subjects**												
								**Single variant on one allele**							**No variant**					

	**NBS (NB2)^+^**	**Ref. Range (μmol/L)**																		
	C14	0–0.42	−	1.41	1.07	4.28	0.54	0.54	0.63	0.36	0.46	0.57	0.48	0.39	0.50	0.37	0.45	0.53	0.72	0.52
	C14:1	0–0.32	−	3.41	1.42	6.35	1.01	0.52	0.63	0.45	0.43	0.49	0.46	0.42	0.48	0.42	0.49	0.43	0.76	0.52
	C14:2	0–0.05	−	0.44	0.16	0.74	0.16	0.06	0.05	0.08	0.03	0.04	0.06	0.05	0.06	0.05	0.06	0.07	0.10	0.04
	C14:1/C2	0–0.014	−	0.200	0.070	0.761	0.039	0.01	0.023	0.018	0.015	0.016	0.017	0.025	0.021	0.026	0.017	0.009	0.020	0.021
	C14:1/C16	0–0.08	−	0.84	0.30	0.50	0.25	0.09	0.10	0.12	0.09	0.07	0.10	0.09	0.09	0.11	0.09	0.06	0.11	0.12
	**Recall (NB2)^+^**	**Ref. range (μmol/L)**																		
	C14	0–0.42	−	0.41	0.29	1.43	−	0.14	−	0.10	−	0.17	−	−	0.06	−	−	−	−	−
	C14:1	0–0.32	−	0.84	0.39	3.39	−	0.05	−	0.07	−	0.07	−	−	0.04	−	−	−	−	−
	C14:2	0–0.05	−	0.18	0.18	0.62	−	0.01	−	0.04	−	0.02	−	−	0.01	−	−	−	−	−
	C14:1/C2	0–0.014	−	0.090	0.062	1.101	−	0.01	−	0.005	−	0.004	−	−	0.004	−	−	−	−	−
	C14:1/C16	0–0.08	−	0.31	0.28	0.92	−	0.04	−	0.06	−	0.02	−	−	0.07	−	−	−	−	−
**Confirmatory testing**	**Acylcarnitines (Chromsys.)^#^**	**Ref. range (μmol/L)**																		
	C14:1	0–0.210	0.52	−	−	−	0.236	−	0.048	−	0.038	−	0.058	0.020	−	0.021	0.028	0.033	0.043	0.020
	C14	0.08–0.5	0.49	−	−	−	0.206	−	0.146	−	0.138	−	0.125	0.058	−	0.092	0.092	0.123	0.194	0.092
	C14:1/C2	0–0.011	0.026	−	−	−	0.020	−	0.0038	−	0.003	−	0.006	0.004	−	0.0023	0.003	0.002	0.003	0.002
	C14:1/C16	0–0.082	0.08	−	−	−	0.054	−	0.0247	−	0.041	−	0.027	0.027	−	0.0364	0.027	0.031	0.031	0.027
	**Organic acids**		−	−	Elevated	Normal	Normal	−	−	−	Normal	−	−	−	−	−	Normal	Normal	−	−
	**NGS (*ACADVL*)**																			
			pat.	lik. pat	lik. pat.	pat.	lik.pat.	−	−	−	−	−	−	−	−	−	−	−	−	−
			lik.pat.	pat.	pat.	pat.	pat.	pat.	pat.	VUS	lik. pat.	pat.	pat.	lik. pat.	−	−	−	−	−	−
	**Enzyme activity**																			
	Lymphocytes^†^		1.26	0.30	0.25	<0.18	0.36	−	−	−	−	1.34	0.91	1.01	−	−	−	−	−	−
	Fibroblasts^‡^		0.27	−	−	−	−	−	−	−	−	−	−	−	−	−	−	−	−	−

All DBSs were collected 48–72 h after birth during a routine newborn screening procedure. *Patient 1 discovered in pilot study prior to start of expanded newborn screening. ^+^Acylcarnitines measured using Perkin Elmer NeoBase2 non-derivatized kit. ^#^Acylcarnitines measured using Chromsystems MassChrom^®^ Amino Acids and Acylcarnitines from Dried Blood derivatized kit; pat., pathogenic, lik. pat., likely pathogenic, VUS, variant of unknown significance (Richards et al., 2015). ^†^For enzyme activity in lymphocytes the reference values are as follows: P1 (1.84–4.80); P2–P4 (2.15–3.79) [nmol min^–1^mgprot^–1^]. ^‡^ or enzyme activity in cultured fibroblasts the reference values are: P1 (1.48–5.24) [nmol min^–1^mgprot^–1^].

**TABLE 3:** Overview of all the *ACADVL* gene variants found.

**PATIENT/CARRIER**	**Nucleotide change (NM_000018.4)**	**Amino acid change (NP_000009)**	**ACMG** classification*	**ACMG criteria met**	**Reported by**
**P1**	c.1837C > T	p.Arg613Trp	Pathogenic	PS3, PP5, PM1, PP2, PM2, PM5, PP3	Strauss et al., 1995
	c.205-8_205-7delCTinsGC	/	Likely pathogenic	PS3, PM2	Hesse et al., 2018
**P2**	c.773T > C	p.Ile258Thr	Likely pathogenic	PS3, PM2, PM1, PP2, PP3	Hesse et al., 2018
	c.1358G > A	p.Arg453Gln	Pathogenic	PS3, PP5, PM1, PP2, PM2, PP3	Andresen et al., 1999
**P3**	c.1077 + 2T > C	/	Pathogenic	PS3, PVS1, PM2, PP5	Andresen et al., 1999
	c.1538C > G	p.Ala513Gly	Likely pathogenic	PS3, PM2, PP2	**Novel**
**P4**	c.1077 + 2T > C	/	Pathogenic	PS3, PVS1, PM2, PP5	Andresen et al., 1999
	c.1358G > A	p.Arg453Gln	Pathogenic	PS3, PP5, PM1, PP2, PM2, PP3	Andresen et al., 1999
**P5**	c.661A > G	p.Ser221Gly	Likely pathogenic	PM2, PP3, PP2	**Novel**
	c.848T > C	p.Val283Ala	Pathogenic	PP5, PM2, PM1, PP2, PP3	Andresen et al., 1996
**P6**	c.1358G > A	p.Arg453Gln	Pathogenic	PS3, PP5, PM1, PP2, PM2, PP3	Andresen et al., 1999
	Not found	–	/	/	/
**P7**	c.848T > C	p.Val283Ala	Pathogenic	PP5, PM2, PM1, PP2, PP3	Andresen et al., 1996
	Not found	–	/	/	/
**P8**	c.416T > C	p.Leu139Pro	VUS	PM2, PP3, PP2	**Novel**
	Not found	–	/	/	/
**P9**	c.1046C > A	p.Ala349Glu	Likely pathogenic	PM1, PP2, PM2, PP3, PP5	**Novel**
	Not found	–	/	/	/
**P10**	c.848T > C	p.Val283Ala	Pathogenic	PP5, PM2, PM1, PP2, PP3	Andresen et al., 1996
	Not found	–	/	/	/
**P11**	c.1837C > T	p.Arg613Trp	Pathogenic	PS3, PP5, PM1, PP2, PM2, PM5, PP3	Strauss et al., 1995
	Not found	–	/	/	/
**P12**	c.1242G > C	p.Glu414Asp	Likely pathogenic	PM2, PM5, PP3, PP2	Hisahara et al., 2015
	Not found	–	/	/	/

Altogether, four novel variants were found. Patients P1–P5 are all compound heterozygous; patients P6–P12 only one heterozygous variant was found. *ACMG classification according to Richards et al. (2015).

**Patient 2** is a 20-months old girl who was first evaluated at 5 days due to a positive NBS result. She is the third child of non-consanguineous parents. Her 6 and 3-years older siblings are healthy. She was born with C-section at 39 weeks of gestation due to the breech presentation. Birth measurements were normal. At the first evaluation, liver transaminases and CK were in the normal range, and the acylcarnitine profile was consistent with VLCADD; the diagnosis was confirmed by low VLCAD enzyme activity (Table 2) and analysis of the *ACADVL* gene (Table 3). She required parenteral hydration with glucose during acute bronchiolitis at 5 months of age and influenza B at 9 months but without overt hypoglycemia. Laboratory tests were mildly elevated aspartate aminotransferase (AST) 2.25 μkat/L (<1.22 μkat/L); alanine aminotransferase (ALT) 1.08 μkat/L (<0.98 μkat/L); CK 24.39 μkat/L (<4.92 μkat/L); myoglobin 116.2 μg/L (<110.0 μg/L). Yearly checkup showed no alterations in structure or function on ECHO of the heart; and normal electrocardiogram (ECG). The US of the abdomen showed no abnormalities. Growth and developmental milestones have been appropriate. The patient was exclusively breastfed for the first month of life. After the first month fat-free milk formula (Basic f, Nutricia, Amsterdam, Netherlands) enriched with 2% MCT oil (2 mL of 100% MCT oil per 100 mL of prepared milk formula), alternately with breastfeeding, was introduced. At the age of 6 months, complementary foods as part of the LCT restricted diet were included. The intake of LCT was limited to 15–30% of total energy intake and at the age of 1 year to 20–30% of total energy intake (Table 1), according to the recommendations (Van Calcar et al., 2020). Dietary requirements for essential fatty acids are being met with walnut oil supplementation. DHA is supplemented when required.

**Patient 3** is a 14-months old boy who was first evaluated at 9 days after screened positive on NBS. After the first and uneventful pregnancy of non-consanguineous parents, he was born at 39 weeks with appropriate birth measurements. On examination, repetitive movements of the head were noticed. The further neurologic evaluation showed axial hypotonia, ataxia, obstructive hypopnea, symmetrical mild hypermetropia, and pale optic papillae were present. The electroencephalogram was normal. Cystic dysplasia of the cerebellum and vermis and hypoplasia of pons were noticed at magnetic resonance scan. A Poretti-Boltshauser syndrome was confirmed. AST, ALT, gamma-glutamyl transferase (GGT), CK, ammonia, and lactate were in the normal range. Acylcarnitine profile was consistent with VLCADD and was confirmed by low enzyme activity (Table 2) and analysis of the *ACADVL* gene (Table 3). The patient did not experience any metabolic crisis to date. ECHO of the heart at the age of 3-months showed no cardiomyopathy. The liver structure was hyperechogenic, without organomegaly. He has a moderate developmental delay; he crawled and sat stably. Growth parameters were normal. Nutritional management of the patient was the same as for patient 2 (Table 1); complementary foods were introduced at 5 months of age. Dietary requirements for essential fatty acids are being met with walnut oil and DHA supplementation.

**Patient 4** is a 1-year-old boy who was first evaluated at 25-days, after the second invitation at a positive NBS. He was born after the first and uneventful pregnancy; delivery was stimulated at 37 weeks of gestation due to detected IUGR (BW 2.3 kg, BL 45 cm). Laboratory tests at first evaluation were mildly elevated AST 1.93 μkat/L (1.22 μkat/L); ALT 0.81 μkat/L (0.98 μkat/L); GGT 2.43 μkat/L (<2.05 μkat/L); CK 1.35 μkat/L (<4.92 μkat/L); ammonia 43 μmol/L (<75 μmol/L); lactate 4.32 mmol/L (1.1–3.5 mmol/L). Acylcarnitine profile was consistent with VLCADD. The diagnosis was later confirmed by non-detectable VLCAD enzyme activity (Table 2) and analysis of the *ACADVL* gene (Table 3). He had no acidosis or hypoglycemia episodes to date; however, he was admitted during respiratory infection with lactate elevation (5.49 mmol/L) at the age of 6 weeks and was given glucose supplementation. US showed enlarged liver (65 mm in MCL) with hyperechogenicity of parenchyma, elastography measured 5.6–6.8 kPa. The developmental assessment showed no delay, and catch-up growth occurred after 2.5 months with the now normal growth rate. Nutritional management of the patient was the same as for patient 3 with the addition of walnut oil to the milk formula (Table 1). The walnut oil and DHA supplementation were later continued.

**Patient 5** is a 5-months old girl who was first evaluated by our NBS metabolic team at 10-days. She had a 5 years old brother, who was followed due to dysplastic kidneys. The mother has arrhythmia. The girl is the second child of non-consanguineous parents. The girl was born at term with normal birth measurements. On examination, poor weight-gain was noticed; the mother reported that she needed to wake the newborn up for breastfeeding. Diet advice was given and weight normalized. AST, ALT, CK, and ammonia were in the normal range. Lactate was mildly elevated [3.84 mmol/L (1.1–3.5 mmol/L)]. Acylcarnitine profile was consistent with VLCADD. The diagnosis was later confirmed by variant analysis of the *ACADVL* gene (Table 3) and by low VLCAD enzyme activity (Table 2). Further evaluation of the heart, and US of the abdomen are still required; but unfortunately, parents’ compliance was poor. The patient was exclusively breastfed for the first 3 weeks of life and then, due to an insufficient weight gain, breastfeeding was supplemented with the regular milk formula. Complementary feeding without mandatory dietary restrictions except for fasting avoidance was introduced at the age of 5 months.

## Results

Results of the first 30,000 newborns screened with extended NBS program revealed 18 subjects with elevated acylcarnitines and acylcarnitine ratios characteristic for VLCADD. Confirmatory testing was done with acylcarnitine analysis from the second DBS card, organic acids analysis from urine, NGS testing, and enzyme activity measurement. Five patients were subsequently confirmed with NGS and enzyme activity testing. Patient 1 was discovered during a pilot study with a different analytical method so no results with the NeoBase2 method are available and patient 1 is therefore omitted from the graphical comparison. For patient 5 confirmatory testing was performed after 10 days after birth and was therefore performed with the Chromsystems method, so there are no results with the NeoBase2 method for recall. NGS confirmatory testing revealed 4 novel *ACADVL* gene variants, 7 heterozygous carriers, and 6 false-positive subjects. Results are presented in Table 2.

### Acylcarnitines Results From the First DBS

**FIGURE 1:**
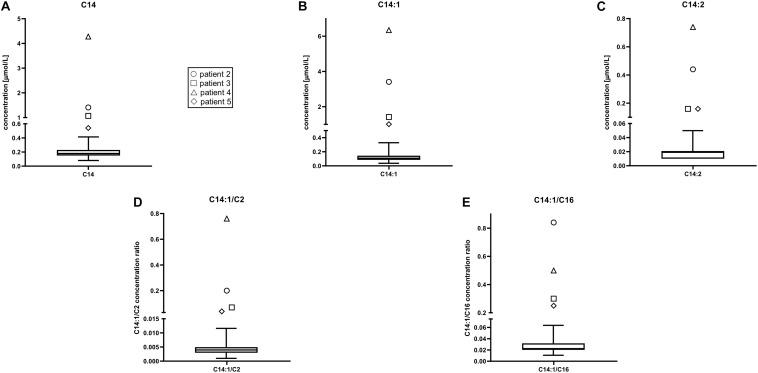
Acylcarnitine concentrations in healthy newborns were compared to those in patients 2 through 5. Patient 1 was not included in the representation because acylcarnitines analysis was performed with different method with different reference values. Patients’ values for all the chosen acylcarnitines were higher than in healthy controls. Box plot and whiskers from data of 17,000 healthy controls. A box represents the 25th and 75th percentile, the line in the box is the median, whiskers are 1st and 99th percentile. C14, tetradecanoylcarnitine (myristoylcarnitine); C14:1, tetradecenoylcarnitine; C14:2, tetradecadienoylcarnitine; C2, acetylcarnitine; C16, hexadecanoylcarnitine. **(A)** C14 concentration (μmol/L), **(B)** C14:1 concentration (μmol/L), **(C)** C14:2 concentration (μmol/L), **(D)** C14:1/C2 concentrations ratio, **(E)** C14:1/C16 concentrations ratio.

Acylcarnitine concentrations and disease-specific acylcarnitines ratios of patients were compared to those of 17,000 healthy newborns. Patients’ (P2–P5) values for all the chosen acylcarnitines and ratios were notably higher compared to values of healthy controls. A box represents the 25th and 75th percentile, the line in the box is the median, whiskers are 1st and 99th percentile (Figure 1). Values of C14:1 in subjects P6–P18 were higher than the highest measured value in healthy newborns. For C14 only P8, P12 and P14 had values below the 99th percentile. For ratio C14:1/C2 only P6 and P16 had values below 99th percentile and for ratio C14:1/C16 only P16 had values below 99th percentile. For C14:2 subjects P7, P9, P10, P12, P14, and P18 had values below the 99th percentile. Patient 1 was analyzed with the Chromsystems method because that was the method of choice in the pilot study.

### Organic Acids

Organic acids in urine were abnormal only in patient 3. Octenedioic, decadienedioic, and hydroxydecanedioic acids were slightly elevated.

### Acylcarnitines Results From Second DBS as Follow-Up

Follow-up DBS samples for patients 2, 3, and 4 came within the first 10 days after birth, and for patient 5 after 10 days. P5 was therefore analyzed with the Chromsystems method. All 4 patients had elevated acylcarnitines and ratios on follow-up. Patient 5 had only elevated C14:1 and C14:1/C2. Among subjects P6–P18, four follow-up DBS samples were analyzed within 10 days after birth, and nine were analyzed later than that. Subjects 6 through 18 showed normal acylcarnitine profile on follow-up from the second card (Table 2).

### Next Generation Sequencing

Patients P1–P5 are compound heterozygotes. In subjects P6–P12 only one heterozygous variant was found and in subjects P13–P18 no variants were found in the *ACADVL* gene. Four novel missense *ACADVL* variants were found. Patients P1 and P2 had one known pathogenic and one known likely pathogenic ACADVL variant. Patient P4 had two known pathogenic *ACADVL* variants. The novel variant in patient P3 (NM_000018.3): c.1538C > G results in p.Ala513Gly substitution (Table 3), with conflicting classification with different prediction algorithms (SIFT, Polyphen 2, MutationTaster, Provean, and CADD). Considering reduced enzyme activity, the variant meets the ACMG criteria for likely pathogenic variant (Richards et al., 2015). The second novel variant in patient P5 c.661A > G results in p.Ala349Glu substitution (Table 3), is classified by most of the prediction algorithms as damaging (SIFT, Polyphen 2, MutationTaster, Provean, and CADD). Considering reduced enzyme activity, the variant meets the ACMG criteria for likely pathogenic variant (Richards et al., 2015). In seven unaffected newborns (P6–P12), only single allele *ACADVL* variants were found in heterozygous state; among these, two were previously unreported. The first one detected in patient P8 c.416T > C results in p.Ala513Gly. Prediction algorithms classify it as pathogenic or likely pathogenic (SIFT, Polyphen 2, MutationTaster, Provean, and CADD) the variant meets ACMG criteria for VUS (Richards et al., 2015). The second variant c.1046C > A was detected in patient P9 and results in p.Ser221Gly. Prediction algorithms classify it as pathogenic or likely pathogenic (SIFT, Polyphen 2, MutationTaster, Provean, and CADD) the variant meets ACMG criteria for likely pathogenic (Richards et al., 2015; Table 3). Enzyme activity.

Enzyme testing on lymphocytes was performed for all patients (P1–P5) with two *ACADVL* variants found with NGS analysis. Results of enzyme activity testing are presented in Table 2. All patients had markedly reduced enzyme activity consistent with genetic findings and confirmatory of the disease. Patient 4 which was the only one with two known pathogenic *ACADVL* variants had the lowest enzyme activity. Patient 1 had enzyme activity measured in both lymphocytes and fibroblasts because the result of enzyme activity in lymphocytes was inconclusive. Enzyme testing was also performed for subjects P10, P11, and P12 which also showed partly reduced enzyme activity in the range of heterozygous carriers.

## Discussion

We reported our initial experiences with expanded NBS using MS/MS and confirmatory NGS for detecting patients with VLCADD. Newborn screening for VLCADD was included in the Slovenian NBS program in 2018. For confirmatory testing of positive results with NGS an in-house panel of causative genes for the diseases included in the NBS program was designed. Additionally, the second DBS card was also analyzed and organic acids measured in the urine of suspected patients.

All acylcarnitines and ratios indicative of VLCADD have been substantially increased in all five confirmed patients (P1–P5). All patients had increased values of C14:1 and other acylcarnitines and ratios on confirmatory test from second DBS card, whereas subjects (P6–P18) in which we did not confirm VLCADD had normal acylcarnitine profile from second card. Organic acids in urine were abnormal only in patient P3 and were as such not a suitable as confirmatory test for VLCADD (Boneh et al., 2006).

Four newborns with specific elevation of acylcarnitines and disease-specific acylcarnitines ratios for VLCADD were confirmed with genetic testing (P2–P5). All patients were compound heterozygotes, with two of them having one novel variant each (c.1538C > G and c.661A > G). One patient was detected through a pilot study before the NBS program was introduced, with specific elevation of acylcarnitines (Smon et al., 2018). Samples in the pilot study were analyzed retrospectively, the average age of the samples at the time of analysis was 8.5 months (from 6 to 11 months). Subsequent NGS *ACADVL* genetic analysis confirmed the diagnosis, the patient was a compound heterozygote. In seven other newborns with positive initial screening results, only single allele variants were found, so the diagnosis was not confirmed. Among these two novel *ACADVL* variants were confirmed. Six newborns with positive screening results were shown to be false positive. These results give us 76.5% of healthy carriers and false positives. Acylcarnitines concentrations of first DBS were higher at four newborns with confirmed disease (C14:1 > 1 μmol/L in all cases) compared to unaffected carriers and false positives. Concentrations at second DBS remained above the reference range for patients 2–4, though lower than the first sample. For newborns where disease was not confirmed results of second testing were not elevated. Still, it is recommended that in the case of positive NBS results suspicious for VLCADD confirmatory testing should be done, as results of second testing can be normal (Hesse et al., 2018). Some authors report that 57% of newborns with positive NBS results for VLCADD are healthy carriers, with one variant in the *ACADVL* gene (Miller et al., 2015). Hesse and coworkers published that 85% of NBS positive cases did not show impaired enzyme function.

There are many causative variants associated with VLCADD; the Human Gene Mutation Database^1^ lists 348 different variants. There is no common variant described as is the case for MCADD, where 54–91% of the alleles carry a common variant (Matsubara et al., 1990; Rhead, 2006). All patients reported in the study were compound heterozygotes. In literature, the variant c.848T > C was described as the most frequent variant causing mild phenotype (Boneh et al., 2006; Miller et al., 2015; Pena et al., 2016; Hesse et al., 2018). In our cohort, it is present on three alleles, in patient 5 in compound heterozygous state together with likely pathogenic variant and in two newborns in heterozygous state. Patient 5 had the lowest acylcarnitine concentrations on NBS of confirmed cases. Variant c.1358G > A was also detected on three alleles. The same variant was described in a homozygous state in two adult patients from Serbia (Fatehi et al., 2020). Enzyme activity was grossly reduced in all five patients (P1–P5) and reduced to about 50% of the lower limit in three carriers (P10–P12) tested, which is in agreement with claims in literature (Leslie et al., 1993; Tajima et al., 2008). Patient 1 discovered in the pilot study, had VLACD activity tested in lymphocytes first, and the result was similar to that of heterozygous carriers. Then sequencing of the *ACADVL* gene was performed in AMC Amsterdam, and two variants were found. The first was a known pathogenic variant and the second was a previously unreported variant with unknown significance. Because of VUS, the genetic result was inconclusive and enzyme testing in fibroblasts was performed to confirm the diagnosis. The result of enzyme activity testing in fibroblasts was markedly reduced enzyme activity and the diagnosis of VLCADD was confirmed. On the three of the heterozygous subjects (P10–P12), we also performed enzyme activity testing. Because there remains a small possibility, that with NGS we would miss a variant on the second allele, and since there is no reliable biochemical testing for VLCADD we decided in 2020 that we would perform enzyme testing on all heterozygous subjects uncovered with NGS.

The estimated VLCADD incidence for the past 2 years was 1:7,500. That is unusually high compared to the literature data with an estimated incidence between 1:30,000 and 1:400,000 live births, and comparable only to the incidence in Saudi Arabia which is known to have a high level of consanguinity. In our families, no consanguinity was reported, and all patients are compound heterozygous. There are large differences in incidence of VLCADD between different populations, which are difficult to compare because of different size and characteristics of populations, details of screening methods, cutoffs, and use of different confirmatory methods (Arnold et al., 2009; Spiekerkoetter et al., 2009; Lindner et al., 2010; Marsden et al., 2021). It is known, that with expanded screening more patients are detected, which is in agreement with our study. There were three MCADD diagnosed cases in the same period, which gives the incidence of 1:10,000. MCADD is the most common inborn error of fatty acid metabolism, with an incidence estimated at 1 in 15,000 live births (Orphanet, 2020: Medium-chain acyl CoA dehydrogenase deficiency; Grosse et al., 2006; Lindner et al., 2010), and our results are in agreement with that. In Southeastern Europe there is currently no NBS for VLCADD in a majority of the countries with exception of Slovenia and Croatia (Groselj et al., 2014a, b, Šmon et al., 2015; Bilandžija et al., 2018; Smon et al., 2018; Lampret et al., 2020; Loeber et al., 2021), which would detect more patients than are diagnosed clinically. To the best of our knowledge, only two adult patients with VLCADD were described so far in Southeastern Europe (Fatehi et al., 2020). Our study presents one of the first descriptions of *ACADVL* variants in Central-Southeastern Europe, detected after implementation of an expanded NBS, showing potentially higher incidence throughout the region as compared to the expected incidence from the literature.

As the treatment strategies for VLCAD include preventing catabolic episodes by providing sufficient energy and nutrient intake, avoiding excessive fasting (especially during illness), and restricting the LCT intake, regular collaboration with an experienced clinical dietitian is required. Medical nutritional therapy for individuals with VLCAD is tailored to the severity of the disorder. Therefore, optimal VLCAD management requires ongoing assessment of clinical as well as nutritional status. Nutrition assessment typically consists of anthropometrics, calculated dietary records with analysis, feeding schedule, and food preferences of the patient. The requirement of special nutrients supplementation (i.e., vitamins A, D, E, DHA, and others) is also assessed. When the patients’ dietary intake does not comply with the recommendations, the patients and their caregivers are invited to an additional dietary consultation with the purpose of dietary adjustment. It is important to monitor the patients regularly and refer them to the hospital when ill, as they can be in danger even with a milder form of VLCADD (Boneh et al., 2006).

The limitation of our study is a relatively small number of NBS results due to only 2 years of expanded NBS. The strength of the study is that all NBS tests and confirmatory diagnostics except enzyme activity measurement are performed in our laboratory. As our laboratory performs routine genetic diagnostics, we can utilize NGS in combination with an in-house panel of genes for confirmatory testing. We have an excellent overview of the age of newborns at the time of sampling, as all the data are automatically transferred from hospitals to laboratory information system (Lampret et al., 2020).

## Conclusion

In the first 2 years of the expanded NBS program in Slovenia altogether about 30,000 newborns were screened. We diagnosed four cases of VLCADD (with the fifth patient diagnosed previously during the pilot study). The estimated VLCADD incidence was 1:7,500; which is much higher than that of MCADD cases in the same period. Our study also provided one of the first descriptions of *ACADVL* variants in Central-Southeastern Europe.

## Data Availability Statement

The raw data supporting the conclusions of this article will be made available by the authors, without undue reservation.

## Ethics Statement

The studies involving human participants were reviewed and approved by the National Medical Ethics Committee (KME: 56/01/14). Written informed consent to participate in this study was provided by the participants’ legal guardian/next of kin.

## Author Contributions

UG and BR conceptualized and designed the study. UG, MZ, and AD performed the clinical work. NL supervised nutritional treatment and collected and analyzed nutritional data. ZR, DP, and MD performed genetic analyses. ZR, VC, DP, BU, and BR carried out the initial NBS analyses and interpreted the results. ZR coordinated and supervised data collection and analysis. BR drafted the initial manuscript. ZR, NL, and AD helped in writing. UG, BR, MD, JK, MZ, TB, and ZR critically reviewed the manuscript for important intellectual content. All authors approved the final manuscript as submitted and agreed to be accountable for all aspects of the work.

## Conflict of Interest

The authors declare that the research was conducted in the absence of any commercial or financial relationships that could be construed as a potential conflict of interest.

## Footnotes

1 https://portal.biobase-international.com
